# The Prevalence and Determinants of Being Offered and Accepting Operational Management Services—A Cohort Study

**DOI:** 10.3390/ijerph18042158

**Published:** 2021-02-23

**Authors:** Adrian Loerbroks, Jessica Scharf, Peter Angerer, Katja Spanier, Matthias Bethge

**Affiliations:** 1 Institute of Occupational, Social and Environmental Medicine, Centre for Health and Society, Faculty of Medicine, University of Düsseldorf, Universitätsstraße 1, 40225 Düsseldorf, Germany; jessica.scharf@hhu.de (J.S.); Peter.Angerer@uni-duesseldorf.de (P.A.); 2 Institute for Social Medicine and Epidemiology, University of Lübeck, Ratzeburger Allee 160, 23562 Lübeck, Germany; Katja.Spanier@uksh.de (K.S.); Matthias.Bethge@uksh.de (M.B.)

**Keywords:** cohort study, Germany, occupational health services, return to work

## Abstract

In Germany, employers are obliged to offer “operational integration management” (OIM) services to employees returning from long-term sick leave. OIM aims to improve employees’ workability and to prevent future sick leave or early retirement. This study examined (i) to what extent OIM services are offered to eligible employees, (ii) to what extent offers are accepted and (iii) the determinants of both outcomes. We used data from a cohort of employees eligible for OIM. Thirty-four potential determinants were assessed in 2013 (i.e., the baseline) using participant reports. In 2015 (i.e., the follow-up), participants were asked (a) whether they had ever been offered OIM services by their employer, and (b) whether they had accepted that offer (i.e., the outcomes). We estimated relative risks by multivariable binomial regression to identify predictors based on backward elimination. In total, 36.0% of the participants were offered OIM services and 77.2% of them accepted that offer. The likelihood of an OIM offer at follow-up was elevated in participants with mental impairment, cancer or long-term absenteeism and increased with organizational justice, neuroticism, and company size. The likelihood of accepting that OIM offer was positively associated with mental impairment and decreased with increasing company size.

## 1. Introduction

The cost of absenteeism is substantial in Western countries. For Germany, for instance, it was estimated at € 136 billion in 2017 [1]. In light of this cost, the ageing of the workforce and shortage of skilled staff in many sectors (e.g., healthcare [2]), it seems crucial to effectively support employees’ return to work (RTW) and their retention in the workforce. Research still needs to produce high-quality evidence though to establish the types of interventions that consistently and successfully contribute to early and sustained RTW [3,4]. It has been suggested that interventions which may be effective are, among other things, carried out early in the RTW process, are cooperatively implemented by multiple types of health professionals and stakeholders (e.g., employers) and focus on workplace adaptations [5,6]. Even if effective interventions are available, their actual implementation is determined by contextual factors, such as RTW management policies, which vary between countries and also between companies in the same country. Overall, the implementation of comprehensive and collaborative RTW programs is often experienced as challenging by the involved parties and stakeholders [7].

In Germany, a specific procedure was introduced in 2004 to facilitate RTW, that is, “operational integration management” (OIM) (in German, betriebliches Eingliederungsmanagement) [8], which has some of the abovementioned characteristics of potentially effective interventions. The overarching aims of OIM are to prevent future (long-term) absenteeism, to improve workability and to ensure that the employee is able to stay at work [8,9]. Employees are legally entitled to OIM services in case they have accumulated six weeks of absenteeism (en bloc or piecemeally) throughout a 12-month period [9]. Eligibility of a given employee is ascertained by employers based on the recorded days of certified sick leave. Employers are then supposed to contact eligible individuals as early as possible to bring OIM as an option to the employee’s attention and to invite her/him for the first meeting [10]. OIM is to be carried out in a case management approach, which implies that the individual needs of the employee are explored and considered. Based on repeated meetings, potentially suitable measures to address those needs are to be identified, implementation plans are agreed upon, the implementation of interventions is monitored, and their effectiveness should be evaluated. The employee may agree to invite different types of internal and external stakeholders to those meetings, for instance, the supervisor, employee representatives (such representatives must be formally involved to some extent), occupational physicians or designated OIM coordinators within the company. In general, OIM measures can address medical, psychological, social and company-related aspects [9], but they usually emphasize work-related modifications. For example, the working hours may be reduced and then gradually increased throughout a defined RTW period, support devices may be provided (e.g., height-adjustable desks), an employee may be transferred to a more suitable workplace within the company, working times or regulations for breaks may be adapted, or training may be provided to enable the returnee to carry out a different set of tasks at work. From a legal perspective, employers are bound to offer OIM services to every eligible employee irrespective of the company size. If employers fail to offer OIM services, a potential legal consequence is that it will be more difficult to dismiss the specific employee due to long-term sickness. However, there are no formal legal penalties for companies not offering OIM, and there is no mandatory documentation of OIM cases within the social welfare system. Employees are not obliged to participate in OIM and may decide to terminate the OIM process at any time. 

It is assumed that OIM offers advantages for employees and employers alike [8]. Employees benefit from the fact that successful OIM may help to reduce their risk of unemployment or early retirement. Advantages to employers are that OIM may contribute to retaining skilled and experienced workers. Further, many of the employers offering OIM services report that absenteeism subsequently decreases, that the commitment among the workforce is enhanced and that there is less layoff due to poor health [11], all of which may contribute to a positive cost–benefit ratio. However, to our knowledge, high-quality evidence documenting the specific benefits of OIM (e.g., in terms of successful RTW or reduced absenteeism) is lacking at present.

Despite generally positive attitudes towards OIM among employers [11] and its potential benefits, the implementation of OIM remains insufficient. This holds particularly true for small companies [11]. To advance insights into the extent to which OIM is offered when it is needed, it seems relevant to use data from employees who are eligible for OIM. Earlier studies [11,12] mostly involved occupational stakeholders or OIM experts, but to a lesser extent eligible employees. Based on a sample of employees entitled to OIM, we therefore set out to (1) estimate the prevalence of receiving an OIM offer, (2) quantify the prevalence of acceptance of that offer and (3) identify predictors of both outcomes based on prospective data. To our knowledge, the latter aim has not been addressed previously. Our findings may help to identify a potential undersupply of occupational RTW support services (i.e., OIM) and insights into the predictors may help to devise interventions to address such a potential supply gap.

## 2. Materials and Methods

### 2.1. Study Population

For the current study, we utilized data from the Third German Sociomedical Panel of Employees (GSPE-III) [13]. The GSPE-III sample was drawn from the register of the Federal German Pension Insurance (GPI). The GPI can grant rehabilitation services to employees to improve their workability and reduce their risk of early retirement. If this is not achieved, the GPI pays disability pensions. Sampling was limited to those who were aged 40–54 years and received sickness absence benefits in 2012 (i.e., one year prior to baseline data collection, see below). In Germany, individuals are eligible for such benefits when they have been on a certified sick leave for a period of at least six weeks due to the same illness. Accordingly, members of our cohort had likewise been entitled to OIM services upon their return to work (see introduction section for eligibility criteria). Baseline data were collected in 2013 by questionnaires, and the cohort was followed up in 2015 and 2017. Information on OIM was only gathered in 2015 and 2017. To ensure that analyses build on an adequate sample size, we combined the 2013 (baseline) and 2015 (follow-up) data for prospective analyses. At baseline, 10,000 questionnaires were sent out and 103 of those could not be delivered. Completed baseline questionnaires were returned by 3294 (33.28%) individuals. Data on age and gender were available for non-responders and we observed that responders were marginally older than non-responders (47.93 vs. 47.25 years) and had a higher proportion of women (53.55% vs. 48.40%). In total, 2233 of the baseline participants (67.79%) returned complete questionnaires at the follow-up in 2015. Those participants differed from non-participants with respect to some sociodemographic characteristics, e.g., they were marginally older, more likely to be female, less likely to speak a mother tongue other than German, and they reported higher educational levels (see Online Resource 1: Table S1). Additional comparisons are presented in the discussion section (see below). For the current analyses, we used data from the 2233 individuals who had completed questionnaires in both 2013 and 2015. We further restricted the sample to those eligible to OIM due to current employment (*n* = 2060) and due to their self-categorization as employees/workers (rather than being in self-employement) (*n* = 2015). Thus, data from a total of 2015 individuals was available to examine the determinants of an OIM offer. Analyses for the second outcome—i.e., acceptance of an OIM offer—were limited to those who reported that they had previously received such an offer (*n* = 691).

The ethics committee of the Hannover Medical School (No. 1730–2013) and the data protection commissioner of the Federal GPI approved this study. The study was registered in the German Clinical Trial Register (No. DRKS00004824).

### 2.2. Determinants at Baseline 

We examined a total of 34 variables as potential determinants (see Table 1 for an overview and Online Resource 2). Determinants were measured using participant reports and covered sociodemographics, health, psychological factors and company size. Sociodemographics included age, sex, speaking a mother tongue other than German (no/yes), being married (no/yes) and the educational level (apprenticeship; technical college; university). Health-related variables comprised accidents and twelve types of illnesses (see Table 1), workability (i.e., the rating of the current workability and disease-related impairment at work as assessed by the German version [14] of the Workability Index [15]), health-related quality of life (i.e., mental and physical summary scores measured by the German version [16] of the SF-36 [17]), absenteeism throughout the previous 12 months (none; up to nine days; 10–24 days; 25–99 days; and 100–365 days) and the number of visits to the general practitioner during the previous twelve months. Psychological factors included social support [18], the effort–reward imbalance (ERI) ratio and overcommitment [19], perceived organizational justice (OJ) [20] and personality traits [21] according to the Big Five model [22] (i.e., neuroticism, extraversion, openness to experience, conscientiousness, and agreeableness). Additional explanations of the psychological constructs are presented in Online Resource 2. Participants further reported the number of employees at their current company. Replies were divided into four categories (< 10; 10–49; 50–249; and 250+ employees) [23]. The first two categories were combined (i.e., < 49 employees) into a single category (labeled “small”) due to small numbers.

### 2.3. Outcomes at Follow-Up

We used the following two outcome variables:

- OIM offer received: participants reported whether they had been offered OIM services by their employers (yes/no).
- OIM offer accepted: those who had provided an affirmative response to the previous question were asked whether they had accepted the OIM offer (yes/no).

### 2.4. Statistical Analyses

We used descriptive statistics to determine sample characteristics and address aims 1 and 2 of our study. To address aim 3 (i.e., the identification of predictors of both outcomes), we assessed relationships between the potential predictors at baseline and the outcomes at follow-up. Doing so, we calculated the relative risk (RR) (or the relative probability) of receiving an OIM offer or of accepting an OIM offer in separate models. As recommended [24], we applied binomial regression models with the log link function in SAS (SAS Institute, Inc., Cary, NC, USA) [25] to estimate RRs and the corresponding 95% confidence intervals (CIs). Whenever possible, we used continuous variables (i.e., z-scores) to operationalize predictors. This approach was employed because the choice of cut-offs was arbitrary for most predictors and because analyses with continuous variables provide more statistical power than analyses with categorized variables. First, we ran separate analyses for each predictor and each outcome and adjusted those models for age and sex. Second, to identify independent determinants, we carried out backward elimination, which is a recommended approach to variable selection [26]. Briefly, in such analyses, the initial model contains all potential predictors. Next, the predictor with the highest *p*-value is removed and the model is rerun. This process is repeated until the model comprises only statistically significant determinants (in our study defined as *p* < 0.05).

## 3. Results

### 3.1. Sample Characteristics and OIM Offer or Acceptance

As shown in Table 1, the participants were, on average, in their late forties (mean = 48.09; standard deviation (SD) = 4.04), slightly more than half were women (55.83%), only few reported that German is not their mother tongue (3.08%) and all educational levels were represented. The three most frequent types of illnesses were musculoskeletal (50.47%), mental (30.12%) and cardiovascular (22.48%). Given their respective potential score ranges, the self-reported levels of workability and health-related impairment at work were rather favorable and the quality of life was at intermediate levels. Social support was high and the ERI was high while overcommitment and OJ showed, on average, intermediate levels. Conscientiousness and agreeableness were the most prominent traits and neuroticism was less pronounced in our sample. Employees from companies with varying sizes were represented with most participants working in large companies. Only 36.01% of the participants reported that they had been offered OIM services by their employer. Whenever such offers were received, three out of four participants reported to have accepted (77.18%).

### 3.2. Determinants of Receiving an OIM Offer

The results from the age- and sex-adjusted analyses are shown and described in Online Resource 3 (Table S2). The final model derived from backward elimination is shown in Table 2. We found weak associations between illnesses and the outcome, which were either positive (i.e., mental impairment and cancer) or inverse (i.e., skin disease). Furthermore, long-term sickness absence predicted an OIM offer (i.e., RR for 100+ sickness absence days versus none = 1.56, 95% CI = 1.22–1.98). Further, we observed rather weak and positive relationships with OJ and neuroticism. For instance, the RR for the OJ z-score was 1.08, which implies that an increase of the OJ score by 1 SD (thus, an increase of 0.87 points according to Table 1 based on the potential score range from 1 to 5 points) was associated with an 8% increase of the probability of being offered OIM services. Company size was the strongest predictor of being offered OIM services (i.e., RR for large versus small companies = 2.44, 95% CI = 2.04–2.91).

### 3.3. Determinants of Accepting an OIM Offer

The results from the age- and sex-adjusted analyses are shown and described in Online Resource 4 (Table S3). Based on backward elimination (Table 3), only two predictors remained in the final model: (i) mental impairment which related to a weakly elevated probability of accepting an OIM offer (RR = 1.12, 95% CI = 1.02–1.21) and (ii) company size suggesting that the probability of accepting an OIM offer was reduced by 13% in medium-sized (RR = 0.87, 95% CI = 0.78–0.96) and by 18% in large companies (RR = 0.82, 95% CI = 0.76–0.89) as compared to small companies.

## 4. Discussion

Our study makes several novel contributions to the existing literature. Firstly, we found that only about one third of the eligible employees were actually offered OIM services, and secondly, that roughly three out of four employees accepted that offer. Further, our study identified several independent predictors of receiving an OIM offer which relate to employees’ health (i.e., mental impairment, skin disease, cancer, and long-term sickness absence), psychosocial characteristics (i.e., OJ and neuroticism) and, in particular, company size. Acceptance of an OIM offer was weakly associated with only two independent predictors; those were mental impairment and the company size.

### 4.1. Interpretation of Findings

The observation that only one third received an OIM offer, but that three quarters decided to accept that offer may highlight considerable unmet needs for support services among employees returning to work form long-term sickness absence. Reasons to decline OIM offers may pertain to the fear of losing one’s job when functional limitations are discussed or concerns regarding data protection [11,27]. Based on the available literature, the high level of acceptance among employees documented by our study can be contrasted with the acceptance levels among employees that relevant organizational stakeholders assume: in a survey addressing OIM for mental illness [12], 60% of the participating OIM experts reported that OIM offers are always or often accepted by eligible employees according to their experience. By contrast, in another survey (mostly involving employee representatives, representatives of employees with severe disability and human resources staff), only 37.1% of the stakeholders assumed that the acceptance of OIM among eligible employees would be high or very high [11]. While those estimates are not readily comparable (e.g., due to larger potential for selection bias in prior surveys [11,12] and sample differences), the current evidence possibly suggests that OIM experts underestimate the acceptance of OIM offers among employees. 

We found that mental impairment and cancer were associated with an increased probability of receiving an OIM offer. In Germany (just like, assumedly, in most other European countries), employees are not obliged to disclose their illness to their employer. Employees may nevertheless disclose their illness or characteristic functional limitations—despite the risk of stigmatization—because disclosure increases the likelihood of receiving more specific support [28]. Upon illness disclosure, employees with mental impairment may be more likely to receive an OIM offer because occupational stakeholders involved in OIM perceive the needs of those employees as particularly demanding [12] and/or because there is increasing awareness, e.g., among supervisors [29], of the importance of their support in that type of RTW process. Employees with cancer may be more likely to receive an OIM offer, because their illness may be perceived as particularly severe and elicits fear in individuals without cancer [30]. Conversely, one may speculate that skin conditions are viewed as less severe or less disabling or that solutions for employees with such conditions are primarily sought through other services (e.g., occupational safety services). This may lower the probability of OIM offers. Individuals with the longest sick leave may be more likely to receive an OIM offer, since long-term absence signals a poor RTW prognosis [31] and therefore employers may be particularly committed to facilitate successful RTW. OJ may positively relate to OIM offers, because OJ is an indicator of employee-oriented workplaces. The positive relationships between neuroticism (i.e., proneness to experience psychological stress [22]) and the probability of an OIM offer may be due to the fact that individuals with high neuroticism are more likely to report somatic symptoms [32]. Alternatively, just as employees with mental impairment, those employees’ needs may be perceived as very demanding, thereby eliciting OIM offers.

Increasing company size was the strongest predictor of future OIM offers. This finding may be explained by the fact that larger companies have more resources: more manpower implies that there is more staff to facilitate the OIM processes, for instance, in terms of administrative tasks, the involvement of specialized OIM teams [11] or in-house occupational health services. Higher financial means imply that larger companies are able to offer a broader set of interventions and more expensive interventions [11], e.g., concerning the adaptation of workplaces. The fact that OIM offers are less likely to occur in small companies should not interpreted as evidence of less support for workers returning from long-term sick leave in small companies. Support in small companies may take a more informal shape and may in fact be particularly strong [11] due to more trustful and closer relationships [33]. Trustful relationships may enable employees to better express their functional limitations, which should result in more suitable interventions in the OIM process.

Regarding acceptance of OIM offers, we found that mental impairment was a weak positive predictor. Possibly, employees with mental impairment are more aware (e.g., due to psychotherapy treatment or counseling) of the importance of support resources to facilitate their coping and are thereby more likely to accept offers. Regarding the company size, we found that the probability of accepting OIM services decreased with company size. This may be because large companies have standardized OIM processes. Therefore, interventions may not be suitably tailored to individual employees who thus decline participation. Furthermore, as mentioned above, trust may be higher in small companies and increases the acceptance of OIM offers (e.g., by reducing the fear of job loss or of misuse of personal data).

### 4.2. Methodological Considerations

The strength of our study is that we were able to draw on data which allowed examining numerous potential predictors. Moreover, our study was based on a prospective design, which usually introduces a temporal sequence between exposures and outcomes and thereby increases confidence in the causality of the observed associations. It needs to be mentioned though that we were unable to establish a temporal sequence in our study with absolute certainty. Due to the wording of the OIM items, we cannot rule out that OIM offers had been made prior to baseline assessments. Further—though it seems unlikely—we cannot rule out that some participants changed their employers throughout the follow-up period. Moreover, our study relied solely on self-reported data, which may be partially misreported. With regard to the OIM items, for instance, we cannot rule out that, in some instances, employees had received an OIM offer but did not recognize it as such (i.e., if the offer was made in an informal way, e.g., in small companies). Another weakness is that our study assessed only a small range of workplace-related data (i.e., company size and OJ perceptions). Further, our response rates and potential selection bias need discussing. The response rate at the follow-up was decent (67.79%). Overall however, only 22.56% of those who received an invitation to complete the baseline questionnaire provided data at both baseline and follow-up assessments for the current analyses (i.e., 2233/9897). Notably though, the extent of potential selection bias is contingent upon the relationship of participation with the exposures of interest, with the outcomes or with the association of those two in a given study [34]. For the current study, the data on exposures (except for age and gender) and outcomes were not available for baseline non-participants. At the follow-up, we observed differences between participants and non-participants with regard to sociodemographic characteristics at baseline (see above and Online Resource 1: Table S1). There was no evidence of a consistent trend though towards better health among follow-up participants as compared to non-participants (including days with sickness absence). With regard to the many psychological variables considered, only social support and effort–reward imbalance seemed to be slightly higher among follow-up participants versus non-participants. Furthermore, at the follow-up, participants were more likely than non-participants to work for a large company. Overall, we thus observed that some of the considered exposures at baseline were associated with follow-up participation. However, potential associations of follow-up participation with the outcomes (i.e., receiving or accepting an OIM offer) remain unknown due to lack of such data. Thus, based on the available data, we are unable to comprehensively examine potential selection bias. It deserves mentioning though that low response rates alone do not necessarily imply selection bias [34]. This notion is supported by previous research involving original data from health surveys [34]. 

We had the opportunity to utilize data from a unique sample of employees eligible for OIM. Due to this special focus, the generalizability of our findings may be limited though: our sample is not representative of the total workforce in Germany. Among other things, this is due to the sampling within a restricted age range (i.e., 40–54 years), the inclusion criterion of having received sickness benefits (i.e., rendering our sample likely less healthy than the general workforce) and the recruitment through the GPI. Workers enrolled in that pension scheme are characterized by a higher socioeconomic status in terms of their educational levels, vocational qualifications and income [35]. Furthermore, they are less frequently exposed to high work-related physical demands and feature a higher proportion of employees (e.g., as opposed to self-employed individuals) compared to the general population [35]. Based on our sampling approach, we can assume good generalizability of our findings specifically to employees who had received sickness benefits. However, while all employees who receive sickness benefits are eligible for OIM, there may be employees who are entitled to OIM, but have not received sickness benefits. These may be employees with several short sick leave episodes that accumulate to six weeks across 12 months and/or who are on a sick leave for varying conditions. Those individuals are not represented in our study.

### 4.3. Recommendation for Research and Practice

Additional studies are needed to corroborate our findings. Preferably, such studies should be based on prospective designs and utilize administrative data whenever possible and suitable (e.g., to define whether an OIM service offer was sent out). Given that company size was the strongest predictor in our study, it seems useful to further explore explanations for that association (see above) and to examine additional workplace-related or economic factors (e.g., the company’s financial means) [33]. Further, characteristics of key players in the OIM process may be of interest. It has been found, for instance, that supervisors’ support of OIM may be higher when they have themselves faced impaired workability [33].

As mentioned above, low availability of OIM offers accompanied by their frequent acceptance suggests a gap in the supply of OIM services. Awareness of this issue needs to be increased among service providers and employers alike. Providers of health or social services (e.g., in rehabilitation clinics) need to inform employees about the fact that they are entitled to OIM, should explain the aims of OIM, the potential procedures and legal rights and support the employee’s decision-making (e.g., whether and how to claim OIM in case that offer is not made). It may be particularly promising to support employees in initiating OIM services themselves, in particular in small companies which may not have established OIM procedures yet or are unaware of OIM [33]. 

Some practice guidelines for employers on how to carry out OIM already exist (e.g., [10]) and awareness of those resources needs to be increased in companies, especially in small companies. Many OIM experts seem to feel though that their company will likely not be able to cope with future OIM cases, in particular due to mental health conditions [12]. Thus, it seems promising to assess the suitability of the available guidelines and how to possibly improve them. For instance, it may be helpful to expand guidelines with illness-specific (e.g., mental illness) or sector-specific (e.g., service sector) information that highlights typical barriers for successful OIM, how to overcome those challenges and context-specific interventions. 

While employers may perceive OIM services to be effective, e.g., in terms of reduced absenteeism [11], experimental evidence is needed to empirically establish such effectiveness. A meta-analysis addressed the potential effectiveness of “RTW coordination programs”, which the authors defined as programs that (i) aim to promote RTW, (ii) build on at least one face-to-face contact between the returnee and a RTW coordinator, (iii) assess the returnee’s needs and devise individualized RTW plans and (iv) whose implementation is managed by a RTW coordinator [3]. Overall, such programs seemed to offer no benefits to workers when compared to usual care, e.g., in terms of successful RTW or reduced absenteeism. Another meta-analysis [4] addressed the effectiveness of workplace-related interventions (e.g., modification of work design, working conditions or environments) to improve RTW and reduce absenteeism and delivered varying findings. It is challenging though to contextualize OIM in light of those meta-analyses and other prior work as comparability is restricted. For example, both meta-analyses examined RTW as the outcome based on studies, which recruited individuals who were on sick leave. However, OIM is offered in the early stages of the actual return to work process. Secondly, except for a consensus on general features of OIM (see Introduction), there is no clear definition of the OIM process and the inclusion criteria of one of the meta-analyses [3] may not cover all cases of OIM as defined in this paper. Overall, high-quality evidence specifically evaluating OIM effectiveness is thus needed. 

## 5. Conclusions

Our study found that only about one third of eligible employees are offered OIM services and that roughly three out of four employees accept that offer. Further, our study identified several independent predictors of receiving or accepting an OIM offer. Future research should confirm our findings and interventions should be modified or devised and evaluated to improve OIM after return to work from long-term absenteeism.

## Figures and Tables

**Table 1:** Baseline characteristics of the study population.

Characteristics		
Age, mean (SD ^a^)		48.09 (4.04)
Sex, *n* (%)	Men	890 (44.17)
	Women	1125 (55.83)
Mother tongue other than German, *n* (%)	No	1952 (96.92)
	Yes	62 (3.08)
Married, *n* (%)	No	718 (35.67)
	Yes	1295 (64.33)
Highest completed educational level, *n* (%)	Apprenticeship	1079 (55.73)
	Technical college	413 (21.33)
	University	444 (22.93)
Accident-related injuries, *n* (%)	No	1605 (79.65)
	Yes	410 (20.35)
Musculoskeletal disease, *n* (%)	No	998 (49.53)
	Yes	1017 (50.47)
Cardiovascular disease, *n* (%)	No	1562 (77.52)
	Yes	453 (22.48)
Respiratory disease, *n* (%)	No	1715 (85.11)
	Yes	300 (14.89)
Mental impairment, *n* (%)	No	1408 (69.88)
	Yes	607 (30.12)
Neurological disease, *n* (%)	No	1656 (82.18)
	Yes	359 (17.82)
Gastrointestinal disease, *n* (%)	No	1776 (88.14)
	Yes	239 (11.86)
Urogenital disease, *n* (%)	No	1851 (91.86)
	Yes	164 (8.14)
Skin disease, *n* (%)	No	1784 (88.54)
	Yes	231 (11.46)
Cancer, *n* (%)	No	1869 (92.75)
	Yes	146 (7.25)
Endocrinological or metabolic disease, *n* (%)	No	1671 (82.93)
	Yes	344 (17.07)
Hematological disease, *n* (%)	No	1946 (96.58)
	Yes	69 (3.42)
Congenital disease, *n* (%)	No	1959 (97.22)
	Yes	56 (2.78)
Workability, mean (SD), potential score range = 0–10		6.80 (2.37)
Disease-related impairment at work, mean (SD), potential score range = 1–6		4.44 (1.40)
Physical health summary score, mean (SD), potential score range = 0–100		45.80 (10.63)
Mental health summary score, mean (SD), potential score range = 0–100		45.00 (12.77)
Self-reported days with sickness absence ^b^, *n* (%)	None	228 (11.37)
	1–9	320 (15.96)
	10–24	299 (14.91)
	25–99	693 (34.56)
	100–365	465 (23.19)
Number of general practitioner visits per year, *n* (%)	0–3	890 (46.48)
	4+	1025 (53.52)
Social support, mean (SD), potential score range = 3–14		9.70 (2.26)
Effort–reward imbalance ratio, mean (SD)		1.37 (0.57)
Overcommitment, mean (SD), potential score range = 6–24		14.94 (2.42)
Organizational justice, mean (SD), potential score range = 1–5		3.09 (0.87)
Neuroticism, mean (SD), potential score range = 3–21		12.37 (4.12)
Extraversion, mean (SD), potential score range = 3–21		14.76 (3.58)
Openness, mean (SD), potential score range = 3–21		14.36 (3.56)
Conscientiousness, mean (SD), potential score range = 3–21		18.65 (2.30)
Agreeableness, mean (SD), potential score range = 3–21		16.72 (2.93)
Company size ^c^, *n* (%)	Small	597 (29.92)
	Medium	445 (22.31)
	Large	953 (47.77)
Operational integration management ^d^ offered, *n* (%)		691 (36.01)
Operational integration management ^d^ accepted, *n* (%)		531 (77.18)

All data in Table 1 were measured at the baseline in 2013, except for the OIM variables, which were taken from the 2015 follow-up. ^a^ SD—standard deviation. ^b^ Note that participants who reported 24 or fewer days of sickness absence were still entitled to operational integration management (OIM): individuals who completed the baseline questionnaire in 2013 received sickness benefits in 2012. Sickness benefits are only granted in case of a sick leave period of at least six weeks with the same illness. If individuals received sickness benefits at any time throughout the year 2012, those six weeks of sick leave must have been accumulated previously (e.g., possibly also in early 2012 or even late 2011). This time period does not necessarily overlap with the days of sick leave reported for the prior period of 12 months at baseline assessments in 2013. Importantly though, all study participants were entitled for OIM upon return to work, because they had received sickness benefits. ^c^ Small—< 49 employees; medium—50–249 employees; large—250+ employees. ^d^ Original labeling of operational integration management (OIM) in German: betriebliches Eingliederungsmanagement (BEM).

**Table 2:** Baseline predictors of the relative risk/probability of being offered operational integration management ^a^ at follow-up; final model based on backward elimination (until *p* < 0.05); thus, all predictors are mutually adjusted.

		RR ^b^	95% CI ^c^
Mental impairment	No	1.0	Ref.
	Yes	1.23	1.08, 1.41
Skin disease	No	1.0	Ref.
	Yes	0.80	0.67, 0.97
Cancer	No	1.0	Ref.
	Yes	1.27	1.08, 1.52
Self-reported days with sickness absence	None	1.0	Ref.
	1-9	0.99	0.74, 1.31
	10-24	1.15	0.88, 1.50
	25-99	1.24	0.97, 1.58
	100-365	1.56	1.22, 1.98
Organizational justice, z-score		1.08	1.03, 1.14
Neuroticism, z-score		1.11	1.04, 1.18
Company size ^d^	Small	1.0	Ref.
	Medium	1.39	1.11, 1.74
	Large	2.44	2.04, 2.91

^a^ Original labeling of operational integration management (OIM) in German: betriebliches Eingliederungsmanagement (BEM); ^b^ RR— risk ratio; ^c^ CI—confidence interval; ^d^ small—< 49 employees; medium—50–249 employees; large—250+ employees.

**Table 3:** Baseline predictors of the relative risk/probability of accepting offered operational integration management ^a^ services at follow-up; final model based on backward elimination (until *p* < 0.05); thus, all predictors are mutually adjusted.

		RR ^b^	95% CI ^c^
Mental impairment	No	1.0	Ref.
	Yes	1.12	1.04, 1.21
Company size ^d^	Small	1.0	Ref.
	Medium	0.87	0.78, 0.96
	Large	0.82	0.76, 0.89

^a^ Original labeling of operational integration management (OIM) in German: betriebliches Eingliederungsmanagement (BEM); ^b^ RR—risk ratio; ^c^ CI—confidence interval; ^d^ small—< 49 employees; medium—50–249 employees; large—250+ employees.

## Data Availability

The data presented in this study are available on request from M.B.

## Supplementary Materials

The following are available online at https://www.mdpi.com/1660-4601/18/4/2158/s1, Online Resource 1: Table S1: Comparison of follow-up non-participants and participants according to baseline characteristics. Online Resource 2: Additional information on the measurement of health-related variables and psychological variables, Online Resource 3: Table S2: Baseline predictors of the relative risk/probability of being offered operational integration management services at the follow-up; age- and sex-adjusted risk ratios, Online Resource 4: Table S3: Baseline predictors of the relative risk/probability of accepting offered operational integration management services at the follow-up; age- and sex-adjusted risk ratios.
