# Supplementary material for: The Prevalence and Determinants of Being Offered and Accepting Operational Management Services—A Cohort Study
Source: Int J Environ Res Public Health. 2021 Feb 23;18(4):2158. doi: 10.3390/ijerph18042158 (PMC7927052; doi:10.3390/ijerph18042158)
Supplement: Supplementary file 1 [file ijerph-18-02158-s001.pdf]

Online Resource 1

Table S1. Comparison of follow-up non-participants and participants according to baseline characteristics.

**Characteristics at Baseline**		**Follow-up Non-Participants**	**Follow-up Participants**	p
		**(*n* = 1061)**	**(*n* = 2233)**	
Age, mean (SD [a])		47.56 (4.24)	48.11 (4.03)	<0.001
Sex, *n* (%)	Men	541 (50.99)	989 (44.29)	<0.001
	Women	520 (49.01)	1244 (55.71)	
Mother tongue other than German, *n* (%)	No	973 (92.67)	2154 (96.55)	<0.001
	Yes	77 (7.33)	77 (3.45)	
Married, n (%)	No	438 (41.71)	815 (36.56)	0.005
	Yes	612 (58.29)	1414 (63.44)	
Highest completed educational level, n (%)	Apprenticeship	586 (60.79)	1207 (56.56)	0.040
	Technical college	195 (20.23)	442 (20.71)	
	University	183 (18.98)	485 (22.73)	
Accident-related injuries, n (%)	No	817 (77.00)	1785 (79.94)	0.053
	Yes	244 (23.00)	448 (20.06)	
Musculoskeletal disease, n (%)	No	576 (54.29)	1099 (49.22)	0.007
	Yes	485 (45.71)	1134 (50.78)	
Cardiovascular disease, n (%)	No	820 (77.29)	1714 (76.76)	0.74
	Yes	241 (22.71)	519 (23.24)	
Respiratory disease, n (%)	No	915 (86.24)	1900 (85.09)	0.38
	Yes	146 (13.76)	333 (14.91)	
Mental impairment, n (%)	No	731 (68.90)	1514 (67.80)	0.53
	Yes	330 (31.10)	719 (32.20)	
Neurological disease, n (%)	No	907 (85.49)	1831 (82.00)	0.013
	Yes	154 (14.51)	402 (18.00)	
Gastrointestinal disease, n (%)	No	949 (89.44)	1966 (88.04)	0.240
	Yes	112 (10.56)	267 (11.96)	
Urogenital disease, n (%)	No	996 (93.87)	2051 (91.85)	0.039
	Yes	65 (6.13)	182 (8.15)	
Skin disease, n (%)	No	941 (88.69)	1981 (88.71)	0.98
	Yes	120 (11.31)	252 (11.29)	
Cancer, n (%)	No	988 (93.12)	2070 (92.70)	0.66
	Yes	73 (6.88)	163 (7.30)	
Endocrinological or metabolic disease, n (%)	No	910 (85.77)	1852 (82.94)	0.039
	Yes	151 (14.23)	381 (17.06)	
Hematological disease, n (%)	No	1040 (98.02)	2158 (96.64)	0.028
	Yes	21 (1.98)	75 (3.36)	
Congenital disease, n (%)	No	1031 (97.17)	2167 (97.04)	0.84
	Yes	30 (2.83)	66 (2.96)	
Work ability, mean (SD), potential score range = 0-10		6.45 (2.64)	6.62 (2.50)	0.073
Disease-related impairment at work, mean (SD), potential score range = 1-6		4.43 (1.40)	4.43 (1.40)	0.99
Physical health summary score, mean (SD), potential score range = 0-100		44.93 (10.98)	45.50 (10.81)	0.17
Mental health summary score, mean (SD), potential score range = 0-100		43.87 (13.36)	44.30 (13.11)	0.39
Self-reported days with sickness absence, *n* (%)	None	112 (12.35)	238 (11.61)	0.57
	1-9	139 (15.33)	328 (16.00)	

	10-24	138 (15.21)	304 (14.83)	
	25-99	290 (31.97)	708 (34.54)	
	100-365	228 (25.14)	472 (23.02)	
Number of annual general practitioner visits, *n* (%)	0-3	481 (49.38)	969 (45.73)	0.059
	4+	493 (50.62)	1150 (54.27)	
Social support, mean (SD), potential score range = 3-14		9.35 (2.30)	9.61 (2.30)	0.002
Effort-Reward-Imbalance ratio, mean (SD)		1.30 (0.56)	1.36 (0.57)	0.003
Overcommitment, mean (SD), potential score range = 6-24		14.86 (2.55)	14.94 (2.42)	0.39
Organizational justice mean (SD) potential score range = 1-5		3.14 (0.88)	3.09 (0.87)	0.19
Neuroticism, mean (SD), potential score range = 3-21		12.55 (4.40)	12.53 (4.16)	0.87
Extraversion, mean (SD), potential score range = 3-21		14.74 (3.70)	14.72 (3.60)	0.88
Openness, mean (SD), potential score range = 3-21		14.49 (3.67)	14.40 (3.59)	0.48
Conscientiousness, mean (SD), potential score range = 3-21		18.59 (2.53)	18.63 (2.35)	0.61
Agreeableness, mean (SD), potential score range = 3-21		16.84 (2.95)	16.73 (2.96)	0.31
Company size [b], , *n* (%)	Small	332 (37.01)	628 (30.86)	<0.001
	Medium	204 (22.74)	448 (22.01)	
	Large	361 (40.25)	959 (47.13)	

[a] SD = standard deviation; [b] Small= <49 employees; medium = 50-249 employees; large = 250+ employees

Online Resource 2

Additional information on the measurement of health-related variables and psychological variables

Health-related variables included:

- Diseases: Reports of accidents and twelve current physician-diagnosed disease groups (see Table 1 in the manuscript), as measured by the Work Ability Index (WAI) [1].
- Work ability, as assessed by two variables from the WAI [1], which were analyzed separately. The first item asked respondents to rate their current work ability compared to the best level of work ability they had ever attained on a scale ranging from 0 (worst) to 10 (best). The second construct was the level of experienced disease-related impairment at work. This construct was assessed by six items that were presented as statements and participants were asked to indicate whether the respective statement applied or not. The resulting total score ranged from 1 to 6 with higher levels indicating less health-related impairment at work.
- Quality of life (QoL) was measured by the German version of the 36-item Short Form Health Survey (SF-36) [2]. We used both the mental health summary score and the physical health summary score. Either score can vary from 0 to 100 and higher scores reflect better health.
- Absenteeism was measured by a WAI item [1] inquiring after the number of days one had been absent from work due to health problems (sickness or health care utilization) throughout the previous 12 months (Response options: none; up to 9 days; 10-24 days; 25-99 days; and 100-365 days).
- Number of visits to the general practitioner as reported during the 12 month prior to data collection.

Psychological factors included:

- Social support was assessed by the 3-item Oslo Social Support Scale [3]. The total score potentially ranges from 3 to 14 whereby higher scores indicated higher social support.
- Effort-Reward-Imbalance (ERI): The ERI model conceptualizes work stress in terms of efforts that exceed one's rewards at work [4,5]. A third component of this model is overcommitment, which is de-

fined as an excessive and intrinsically motivated work drive [5]. We used a abbreviate questionnaire [6] and calculated the ER ratio as recommended [6]. Higher scores indicated more work stress in terms of the ERI model. Overcommitment (measured by six items) was used as a separate variable, that is, a sum score potentially varying from 6 to 24 whereby higher scores reflect more overcommitment.

- Organizational justice (OJ): OJ is a work stress concept that emphasizes the role of fairness perceptions at workplaces [7,8]. OJ was assessed by an established 13-item German-language instrument [9]. The level of agreement with each item was to be expressed on a 5-point scale. We calculated a mean score across all items (potential range: 1-5). Higher scores indicate higher perceived OJ.
- Personality: The German-language Big Five Inventory was employed to capture personality traits [10]. The five key personality traits of the Big Five model [11] are measured by three statements each and agreement is reported using a 7-point response format ("1= does not apply at all" to "7=fully applies"). The five measured traits are labeled neuroticism (i.e. proneness to experiencing psychological stress), extraversion (i.e. the tendency to be socialable, outgoing and energetic), agreeableness (i.e. the tendency to be cooperative and not competitive towards others), conscientiousness (i.e., proneness to be self-organized, self-disciplined and to aim for achievement), and openness to experience (i.e., curiosity and a preference for novelty rather than routines).

Company size: Participants reported the number of employees at their current company. Replies were provided by four categories (<10; 10-49; 50-240, and 250+ employees). The first two categories were combined (i.e. < 49 employees) into a single category (labeled "small") due to low numbers.

References

1. Tuomi K, I.J., Jahkola A, Katajarinne L, Tulkki A. *Arbeitsbewältigungsindex. Work Ability Index*; Wirtschaftsverlag NW, Verlag für Neue Wissenschaft: Bremerhaven, Germany, 2001.

2. Morfeld, M.; Kirchberger, I.; Bullinger, M. *SF-36 Fragebogen zum Gesundheitszustand: Deutsche Version des Short Form-36 Health Survey [SF-36 questionnaire on the health status: German version of the Short Form-36 Health Survey]*; Hogrefe: Göttingen, Germany, 2011.

3. Dalgard, O.S.; Dowrick, C.; Lehtinen, V.; Vazquez-Barquero, J.L.; Casey, P.; Wilkinson, G.; Ayuso-Mateos, J.L.; Page, H.; Dunn, G.; Group, O. Negative life events, social support and gender difference in depression: a multinational community survey with data from the ODIN study. *Soc Psychiatry Psychiatr Epidemiol* **2006**, *41*, 444-451.

4. Siegrist, J.; Starke, D.; Chandola, T.; Godin, I.; Marmot, M.; Niedhammer, I.; Peter, R. The measurement of effort-reward imbalance at work: European comparisons. *Soc Sci Med* **2004**, *58*, 1483-1499.

5. Siegrist, J. Effort-reward imbalance and health in a globalized economy. *SJWEH* **2008**, *6 (Suppl)*, 163-168.

6. Siegrist, J.; Wege, N.; Puhlhofer, F.; Wahrendorf, M. A short generic measure of work stress in the era of globalization: effort-reward imbalance. *Int Arch Occup Environ Health* **2009**, *82*, 1005-1013.

7. Colquitt, J.; Conlon, D.; Wesson, M.; Porter, C.; Ng, K. Justice at the millennium: a meta-analytic review of 25 years of organizational justice research. *J Appl Psychol* **2001**, *86*, 425-445.

8. Elovainio, M.; Kivimaki, M.; Vahtera, J. Organizational justice: evidence of a new psychosocial predictor of health. *Am J Public Health* **2002**, *92*, 105-108.

9. Spanier, K.; Radoschewski, F.M.; Gutenbrunner, C.; Bethge, M. Direct and indirect effects of organizational justice on work ability. *Occup Med (Lond)* **2014**, *64*, 638-643.

10. Rammstedt, B.; John, O. Short version of the Big Five Inventory (BFI-K): Development and validation of an economic inventory for assessment of the five factors of personality. *Diagnostica* **2005**, *51*, 195-206.

11. Costa, P.T., Jr. ; McCrae, R.R. *Revised NEO Personality Inventory (NEO-PI-R) and NEO Five-Factor Inventory (NEO-FFI) manual*; Psychological Assessment Resources: Odessa, USA,1992.

Online Resource 3

Table S2. Baseline predictors of the relative risk/probability of being offered operational integration management[a] services at follow-up; age-and sex-adjusted risk ratios

		RR[b]	95% CI[c]
Age (continuous)		1.01	0.99, 1.02
Sex	Men	1.0	Ref
	Women	1.02	0.91, 1.15
Mother tongue other than German	No	1.0	Ref
	Yes	1.01	0.72, 1.42
Married	No	1.0	Ref
	Yes	1.03	0.91, 1.17
Highest completed educational level	Apprentice-ship	1.0	Ref
	Technical college	0.99	0.85, 1.16
	University	1.03	0.89, 1.20
Accident-related injuries	No	1.0	Ref
	Yes	0.91	0.78, 1.06
Musculoskeletal disease	No	1.0	Ref
	Yes	1.10	0.97, 1.24
Cardiovascular disease	No	1.0	Ref
	Yes	1.13	0.99, 1.30
Respiratory disease	No	1.0	Ref
	Yes	0.93	0.78, 1.10
Mental impairment	No	1.0	Ref
	Yes	1.40	1.25, 1.58
Neurological disease	No	1.0	Ref
	Yes	1.08	0.93, 1.25
Gastrointestinal disease	No	1.0	Ref
	Yes	1.20	1.01, 1.41

Urogenital disease	No	1.0	Ref
	Yes	1.10	0.89, 1.35
Skin disease	No	1.0	Ref
	Yes	0.88	0.72, 1.08
Cancer	No	1.0	Ref
	Yes	1.28	1.05, 1.55
Endocrinological or metabolic disease	No	1.0	Ref
	Yes	1.12	0.97, 1.31
Hematological disease	No	1.0	Ref
	Yes	1.27	0.98, 1.66
Congenital disease	No	1.0	Ref
	Yes	1.57	1.21, 2.03
Work ability z-score		0.89	0.85, 0.94
Disease-related impairment at work z-score		0.87	0.83, 0.92
Physical health summary z-score		0.90	0.88, 0.95
Mental health summary z-score		0.90	0.85, 0.95
Self-reported days with sickness absence	None	1.0	Ref
	1-9	0.99	0.74, 1.32
	10-24	1.30	0.99, 1.72
	25-99	1.36	1.06, 1.73
	100-365	1.85	1.45, 2.35
Number of annual general practitioner visits	0-3	1.0	Ref
	4+	1.28	1.13, 1.46
Social support z-score		1.01	0.96, 1.07
Effort-Reward-Imbalance ratio z-score		1.04	0.99, 1.11
Overcommitment z-score		1.11	1.04, 1.18
Organizational justice z-score		1.00	0.94, 1.06
Neuroticism z-score		1.12	1.05, 1.19
Extraversion z-score		0.95	0.90, 1.01
Openness z-score		0.95	0.90, 1.01
Conscientiousness z-score		0.93	0.88, 0.98

Agreeableness z-score		1.02	0.96, 1.08
Company size[d]	Small	1.0	Ref
	Medium	1.38	1.10, 1.72
	Large	2.47	2.07, 2.94

[a] Original labeling of operational integration management (OIM) in German: "Betriebliches Eingliederungsmanagement" (BEM)

[b] RR= Risk ratio

[c] CI = Confidence interval

[d] Small= <49 employees; medium = 50-249 employees; large = 250+ employees

Description Table S2:

Sociodemographic variables showed no meaningful associations with receiving an OIM services offer. Among the health-related variables, mental impairment, gastrointestinal disease, cancer and congenital disease were associated with the outcome (i.e., relative risks ranging from 1.2 to 1.6). Better work ability, decreasing levels of health-related impairment at work as well as better physical and mental quality of life z-scores were associated with a slightly reduced probability of being offered OIM services (all RRs = 0.9). Further, increasing sickness absence predicted receiving an OIM services offer (e.g. RR for 100+ days versus none= 1.9, 95%CI=1.5-2.4) which also held true for the number of general practitioner visits (RR for 4+ visits versus less=1.3, 95%CI=1.1-1.5). With regard to psychological variables, we found that the likelihood of being offered OIM was positively associated overcommitment and neuroticism, but inversely associated with conscientiousness. Company size showed the strongest relationship with the outcome, for instance, the probability of an OIM service offer was 2.5-fold higher in large companies (95%CI=2.1-2.9) as compared to small companies.

Online Resource 4

Table S3. Baseline predictors of the relative risk/probability of accepting offered operational integration management[a] services at follow-up; age-and sex-adjusted risk ratios

		RR[b]	95% CI[c]
Age (continuous)		0.99	0.98, 1.00
Sex	Men	1.0	Ref
	Women	1.05	0.97, 1.14
Mother tongue other than German	No	1.0	Ref
	Yes	0.89	0.69, 1.19
Married	No	1.0	Ref
	Yes	0.97	0.89, 1.05
Highest completed educational level	Apprenticeship	1.0	Ref
	Technical college	1.01	0.92, 1.12
	University	0.97	0.87, 1.08
Accident-related injuries[c]	No	1.0	Ref
	Yes	0.97	0.87, 1.08
Musculoskeletal disease	No	1.0	Ref
	Yes	1.09	1.00, 1.18
Cardiovascular disease	No	1.0	Ref
	Yes	1.01	0.92, 1.11
Respiratory disease	No	1.0	Ref
	Yes	1.03	0.92, 1.16
Mental impairment	No	1.0	Ref
	Yes	1.15	1.06, 1.24
Neurological disease	No	1.0	Ref
	Yes	1.01	0.92, 1.12
Gastrointestinal disease	No	1.0	Ref
	Yes	1.00	0.89, 1.13
Urogenital disease	No	1.0	Ref

	Yes	1.13	1.01, 1.25
Skin disease	No	1.0	Ref
	Yes	1.05	0.93, 1.19
Cancer	No	1.0	Ref
	Yes	0.93	0.79, 1.09
Endocrinological or metabolic disease	No	1.0	Ref
	Yes	0.89	0.79, 1.01
Hematological disease	No	1.0	Ref
	Yes	0.90	0.72, 1.13
Congenital disease	No	1.0	Ref
	Yes	1.04	0.88, 1.23
Work ability z-score[d]		0.96	0.92, 0.99
Disease-related impairment at work z-score[e]		0.95	0.92, 0.99
Physical health summary z-score[f]		1.00	0.96, 1.04
Mental health summary z-score[g]		0.95	0.91, 0.98
Self-reported days with sickness absence	None	1.0	Ref
	1-9	1.19	0.96, 1.47
	10-24	1.15	0.93, 1.42
	25-99	1.15	0.95, 1.41
	100-365	1.23	1.01, 1.49
Number of annual general practitioner visits[h]	0-3	1.0	Ref
	4+	1.06	0.97, 1.16
Social support z-score[i]		0.97	0.93, 1.02
Effort-Reward-Imbalance ratio z-score[j]		1.00	0.96, 1.04
Overcommitment z-score[k]		1.05	1.01, 1.09
Organizational justice z-score[l]		1.01	0.97, 1.05
Neuroticism z-score[m]		1.05	1.01, 1.10
Extraversion z-score		0.95	0.92, 0.99
Openness z-score		1.00	0.96, 1.04
Conscientiousness z-score		1.00	0.96, 1.04
Agreeableness z-score		1.03	0.99, 1.08

Company size[d]	Small	1.0	Ref
	Medium	0.88	0.79, 0.99
	Large	0.81	0.74, 0.87

[a] Original labeling of o*perational integration management (OIM) in German:* "Betriebliches Eingliederungsmanagement" (BEM)

[b] RR= Risk ratio

[c] CI = Confidence interval

[d] Small= <49 employees; medium = 50-249 employees; large = 250+ employees

Description Table S3:

Several determinants were observed in age-and-sex-adjusted analyses; overall though associations were very weak (i.e., significant RRs varied between 0.8 and 1.2). The probability of accepting an OIM was elevated in case of mental impairment, urogenital disease, long-term sickness absence, and increased with neuroticism. The probability was reduced with increasing work ability, decreasing health-related impairment at work, and increasing extraversion. Importantly, increasing company size appeared to be associated with a slightly *reduced* probability of accepting an OIM offer.
